# The Lost Art of Hospital Ventilation

**Published:** 1924-10

**Authors:** S. S. Goldwater

**Affiliations:** DIRECTOR OF MOUNT SINAI HOSPITAL, NEW YORK


### THE LOST ART OF HOSPITAL VENTILATION.

**By S. S. GOLDWATER, M.D., DIRECTOR OF MOUNT SINAI HOSPITAL, NEW YORK.**

[Ventilation is so important a matter in hospitals, and artificial methods have so often been found unsatisfactory, that our readers will be interested in the following extracts from a breezy article on the subject by the most distinguished American hospital authority, which we quote, by kind permission, from *The Modern Hospital*, of Chicago.]

Some twenty years ago the ward buildings of a prominent hospital in New York were equipped with supply and exhaust fans. The wards in question had high ceilings, broad double-hung windows with transoms, and an excellent south-west exposure; they opened into wide-windowed corridors which, in turn, communicated with open stairways serving as ventilating flues. In the wards themselves the space allowance per bed was fully 1,800 cubic feet, or, approximately, 50 per cent. above the prevailing standard. All of the conditions favoured abundant natural ventilation. Twenty years have passed, and the costly mechanical ventilating equipment of these wards has never once been operated!

### "Where Rubbish May be Shot."

Some years ago a pile of sheet-iron ducts, grimy with age, began to make its appearance in the yard of another hospital. Week after week the pile increased until its proportions were truly amazing. Inquiry elicited the information that the hospital had long since abandoned its mechanical ventilating system, that the ducts with which the building was encumbered were growing increasingly foul, and that, as they were performing no service, it was deemed best to remove and destroy them; and destroyed they were, with a vigour and a thoroughness that would have done credit to a Joshua in Biblical times, to a Jenghis Khan in the twelfth century, or to a Teutonic apostle of ruthless warfare in our own era of moral apathy and scientific efficiency.

### Broken Windows Supplement the System.

A third instance of a quite different character comes to mind. It is that of a small country hospital in which a complete mechanical ventilating system was placed. In this case the ventilating engineer, supported by the management of the hospital, proposed to take no chances. The fans were promptly set in operation when the hospital service was inaugurated and all of the windows were closed and securely fastened in order to prevent any possible interference with the operation of the double supply and exhaust system. I happened to visit this hospital during its memorable first winter, and as I went about the building I noticed several broken window panes. The superintendent explained that these were due to failure on the part of nurses and others to appreciate the excellence of the ventilating equipment. The volume of air that was being moved through the building by the supply and exhaust fans was all that the accepted physiologic rules of the period demanded, but the inmates of the hospital were exasperated at the sight of closed and locked windows, and nearly every day window panes were broken and had to be replaced.

### Importance of Outside Windows.

It is evident, then, that allowance for average human conduct or behaviour should be made by the ventilating engineer who makes a mechanical ventilating system available for use. One of the cases cited above shows that a potentially effective mechanical system may be placed in a hospital and may never be used. The designer of such a system can never be sure, in advance, of its actual operation. Indeed, since it is now known that a majority of the ventilating fans that are placed in hospitals are not used as they were intended to be used, it is reckless, it is almost foolish to plan a hospital building in which important rooms have no outside windows. I recall the instance of a crowded dispensary waiting hall which is surrounded by examining rooms through which fresh air cannot be admitted during the winter months without the undue exposure of patients. In this case the original plenum system upon which the engineer relied has long since been discarded, and the present conditions can easily be imagined.

### Natural Ventilation Not Replaced.

It is in connection with private rooms, wards, out-patient departments, and dormitories that one most frequently finds abandoned mechanical ventilating systems, and it is, therefore, wise to plan these sections of hospitals on the assumption that natural ventilation may at any time be resorted to, notwithstanding the installation of fans and ducts. The mechanical installation then becomes an additional means, not the sole means, of ventilation, and since it is regarded only as a potential auxiliary not quite dependable under the circumstances, there is every reason to use one's best endeavours to make the conditions as favourable as possible for natural ventilation. This means that window spaces should be ample; that in the case of sliding sash, raised stools or lowered sub-sills should be employed, so that the lower sash may be slightly raised, affording an inlet for fresh air at the meeting rail, without creating a draught beneath the bottom rail; that a second slight adjustable opening, available in cold weather, should be provided in the form of a transom or by inserting a small adjustable ventilator in the bottom rail of the lower sash. When these precautions are taken it is possible to keep the air of a ward fairly fresh throughout the winter without serious discomfort to any occupant.

### But!

Having pointed out that a mechanical ventilating system may be installed and not used, I am bound to acknowledge that the same fate may befall a proposed system of natural ventilation. Is it likely, for example, that a large ward having windows on both sides, with every imaginable device to promote window ventilation, will be freely aired at night, or kept in the winter months at a temperature which is comfortable for patients lying under blankets, if the desk at which the night nurse sits for hours to do her clinical charting is in the open ward?

